# Supplementary material for: Scanning electron microscopy (SEM) reveals high diversity of setae on the hind tibiae and basitarsi of Peruvian Stingless Bees (Apidae: Meliponini)
Source: PeerJ. 2025 Oct 9;13:e19749. doi: 10.7717/peerj.19749 (PMC12515428; doi:10.7717/peerj.19749)
Supplement: Supplemental Information 1 — TL: Tibia length. TB: Tibia breadth. BL: Basitarsus length. BW: Basitarsus breadth. Units of measure: µm. [file peerj-13-19749-s001.docx]

|  | *M.* cf. *eburnea* | | *P. testacea* | | *S.* cf. *latitarsis* | | *T.*  *dallatorreana* | | *T.* cf. *atomaria* | | *T.* cf. *hypogea* | | *Lestrimelitta* sp. | |
| --- | --- | --- | --- | --- | --- | --- | --- | --- | --- | --- | --- | --- | --- | --- |
| **Hind tibia (TL\|TB)** | 3079,3 | 1062,0 | 2234,7 | 1094,8 | 1538,5 | 620,3 | 2501,9 | 846,4 | 862,9 | 265,8 | 2568,7 | 764,2 | 1683,4 | 560,2 |
| **Hind basitarsus (BL\|BW)** | 1263,8 | 646,0 | 1122,4 | 601,1 | 1080,0 | 495,3 | 908,0 | 489,0 | 247,2 | 176,6 | 965,0 | 547,7 | 878,9 | 288,9 |
