# Supplementary material for: Scanning electron microscopy (SEM) reveals high diversity of setae on the hind tibiae and basitarsi of Peruvian Stingless Bees (Apidae: Meliponini)
Source: PeerJ. 2025 Oct 9;13:e19749. doi: 10.7717/peerj.19749 (PMC12515428; doi:10.7717/peerj.19749)
Supplement: Supplemental Information 2 — DM: Distal margin. SZ: Supraglabrate zone. AZ: Apicalglabrate zone. –: Absent. [file peerj-13-19749-s002.docx]

| **Species** | **Shape** | **Corbicula** | **DM** | **SZ** | **AZ** | **Clivulus** | **Cuticular sculpture** |
| --- | --- | --- | --- | --- | --- | --- | --- |
| *Lestrimelitta* sp. | Club-shaped | Flat | Straight | Reduced | – | – | Smooth |
| *M.* cf. *eburnea* | Triangular | Concave | Straight, then arched. Distal acute angle. | Narrow strip | – | – | Fundus with long, broad imbrications forming parallel stripes. |
| *P. testacea* | Spoon-like | Concave | Arched | Narrow strip | Wide | – | Short, irregular, interrupted imbricatures. |
| *S.* cf. *latitarsis* | Triangular | Concave | Protuberance in the middle. Distal acute angle. | Absent | Wide | – | Long, wide imbricatures, forming parallel lines. |
| *T. dallatorreana* | Hockey stick | Slightly concave | Straight | Wide | Wide | Abrupt | Smooth; weak lozenge-shaped imbrications near distal margin. |
| *T.* cf. *hypogea* | Club-shaped | Flat | Straight | Wide | Wide | Abrupt | Smooth |
| *T.* cf. *atomaria* | Drop-shaped | Slightly concave | Sinuous, ending in a distal acute angle. | Reduced | Reduced | – | Strongly imbricated, with imbricatures shaped as polygonal lozenges. |
