# Supplementary material for: Scanning electron microscopy (SEM) reveals high diversity of setae on the hind tibiae and basitarsi of Peruvian Stingless Bees (Apidae: Meliponini)
Source: PeerJ. 2025 Oct 9;13:e19749. doi: 10.7717/peerj.19749 (PMC12515428; doi:10.7717/peerj.19749)
Supplement: Supplemental Information 3 — RM: Retrodorsal margin. DB: Distal margin. da: Distal angle. [file peerj-13-19749-s003.docx]

| **Species** | **Shape** | **RM** | **DB** | **da** | **Cuticular sculpture** |
| --- | --- | --- | --- | --- | --- |
| *Lestrimelitta* sp. | Stick-shaped | Straight | Straight | Right | Smooth. |
| *M.* cf. *eburnea* | Rectangular | Arched | Arched | Acute | Imbricated near proventral margin, smooth elsewhere. |
| *P. testacea* | Concave polygonal | Straight at first, then arched | Notched with rounded edges | Obtuse | Imbricated near proventral margin, smooth elsewhere. |
| *S.* cf. *latitarsis* | Parrot-beak shape | Prominently arched | Straight | Acute | Imbricated near proventral margin, smooth elsewhere. |
| *T. dallatorreana* | Rectangular | Slightly arched | Straight | Right | Smooth. |
| *T.* cf. *hypogea* | Rectangular | Slightly arched | Slightly arched | Acute | Smooth. |
| *T.* cf. *atomaria* | Concave polygonal | Slightly arched | Notched with rounded edges | Obtuse | Imbricated, becoming smooth towards the retrodorsal margin. |
