# Supplementary material for: Scanning electron microscopy (SEM) reveals high diversity of setae on the hind tibiae and basitarsi of Peruvian Stingless Bees (Apidae: Meliponini)
Source: PeerJ. 2025 Oct 9;13:e19749. doi: 10.7717/peerj.19749 (PMC12515428; doi:10.7717/peerj.19749)
Supplement: Supplemental Information 4 — For each species, a range of lengths (in µm) of the setae is provided, indicating the shortest and longest. -: Absent. [file peerj-13-19749-s004.docx]

| **Species** | **SETAL TYPES** | | | | | | | | | | | |
| --- | --- | --- | --- | --- | --- | --- | --- | --- | --- | --- | --- | --- |
|  | **Simple setae** | | | | | **Branched setae** | | | | | | |
|  | **ns** | **ts** | **hs1** | **hs2** | **hs3** | **sp** | **pe1** | **pe2** | **pl** | **de1** | **de2** | **de3** |
| *Lestrimelitta* sp. | 22,3-71,0 | 59,7-112,1 | - | - | - | 122,6-208,5 | - | 105,9-131,7 | 54,7-59,3 | 50,3-49,2 | - | - |
| *M.* cf. *eburnea* | 35,7-84,9 | 118,3-222,2 | 86,5-130,0 | - | 77,2-755,4 | - | - | - | - | - | 79,1-106,2 | 111,5-145,5 |
| *P. testacea* | 32,5-58,5 | 89,6-124,5 | 68,4-131,7 | 1047-263,24 | 89,5-896,7 | 196,7-518,2 | - | - | - | - | - | 64,7-106,8 |
| *S.* cf. *latitarsis* | 24,5-29,3 | 39,1-94,3 | 74,8-139,6 | - | 43,8-333,7 | 41,9-64,6 | - | 41,9-55,5 | 47,3-82,8 | 24,8-25,0 | - | - |
| *T. dallatorreana* | 88,0-96,0 | 202,3-310,9 | 29,7-48,8 | - | 33,3-384,5 | 56,6-264,3 | 126,7-172,8 | 104,1-435,8 | - | 42,2-92,1 | - | - |
| *T.* cf. *hypogea* | 68,7-73,6 | 116,4-129,0 | - | 274,5-289,5 | 75,-392,3 | 154,0-292,4 | 103,5-116,1 | 38,7-262,1 | - | 33,7-54,7 | - | 62,1-77,3 |
| *T.* cf. *atomaria* | 9,1-15,0 | 40,3-48,7 | - | - | 30,9-270,1 | - | - | - | - | 16,2-40,1 | 7,2-24,1 | - |
