# Supplementary material for: Scanning electron microscopy (SEM) reveals high diversity of setae on the hind tibiae and basitarsi of Peruvian Stingless Bees (Apidae: Meliponini)
Source: PeerJ. 2025 Oct 9;13:e19749. doi: 10.7717/peerj.19749 (PMC12515428; doi:10.7717/peerj.19749)
Supplement: Supplemental Information 5 — For each of these structures, either the range between the shortest and longest seta observed is given, or the mean length ± standard deviation (SD) based on three representative setae are reported if no appreciable variation in length was found. Units of measure: µm. -: Absent. [file peerj-13-19749-s005.docx]

|  | *M.* cf. *eburnea* | *P. testacea* | *S.* cf. *latitarsis* | *T.*  *dallatorreana* | *T.* cf. *atomaria* | *T.* cf. *hypogea* | *Lestrimelitta* sp. |
| --- | --- | --- | --- | --- | --- | --- | --- |
| **Inferior**  **parapenicillum** | 170,1-371,6 | 107,9-464,2 | 128,2-151,7 | 388,7±30,0 | 63,7±6,5 | 130,3±27,0 | 171,3±25,6 |
| **Penicillum** | 269,7-621,0 | 220,2-465,2 | 142,7-279,6 | 228,0-439,8 | 57,9-184,4 | 177,5-268,5 | - |
| **Superior**  **parapenicillum** | 565,0±85,6 | 335,8±88,2 | 295,6±78,7 | 380,0±22,9 | 205,9±31,6 | - | - |
| **Rastellum** | 96,3-298,3 | 72,9-181,2 | 114,2-138,5 | 109,0-158,8 | 36,5-72,2 | 111,1±3,07 | - |
| **Keirotrichia** | 61,9±7,1 | 61,7±3,8 | 32,3±4,9 | 108,5±24,8 | 29,0±2,2 | 49,3±3,9 | 61,2±2,0 |
| **Pollen brush** | 84,8-349,8 | 142,0-290,3 | 51,6-106,9 | 88,8-563,2 | 16,6-52,2 | 46,6-174,9 | 64,8-121,7 |
| **Sericeus area** | - | - | - | 18,6-21,8 | - | 19,8-20,5 | - |
