# Supplementary figures and images for: Scanning electron microscopy (SEM) reveals high diversity of setae on the hind tibiae and basitarsi of Peruvian Stingless Bees (Apidae: Meliponini)
Source: PeerJ. 2025 Oct 9;13:e19749. doi: 10.7717/peerj.19749 (PMC12515428; doi:10.7717/peerj.19749)

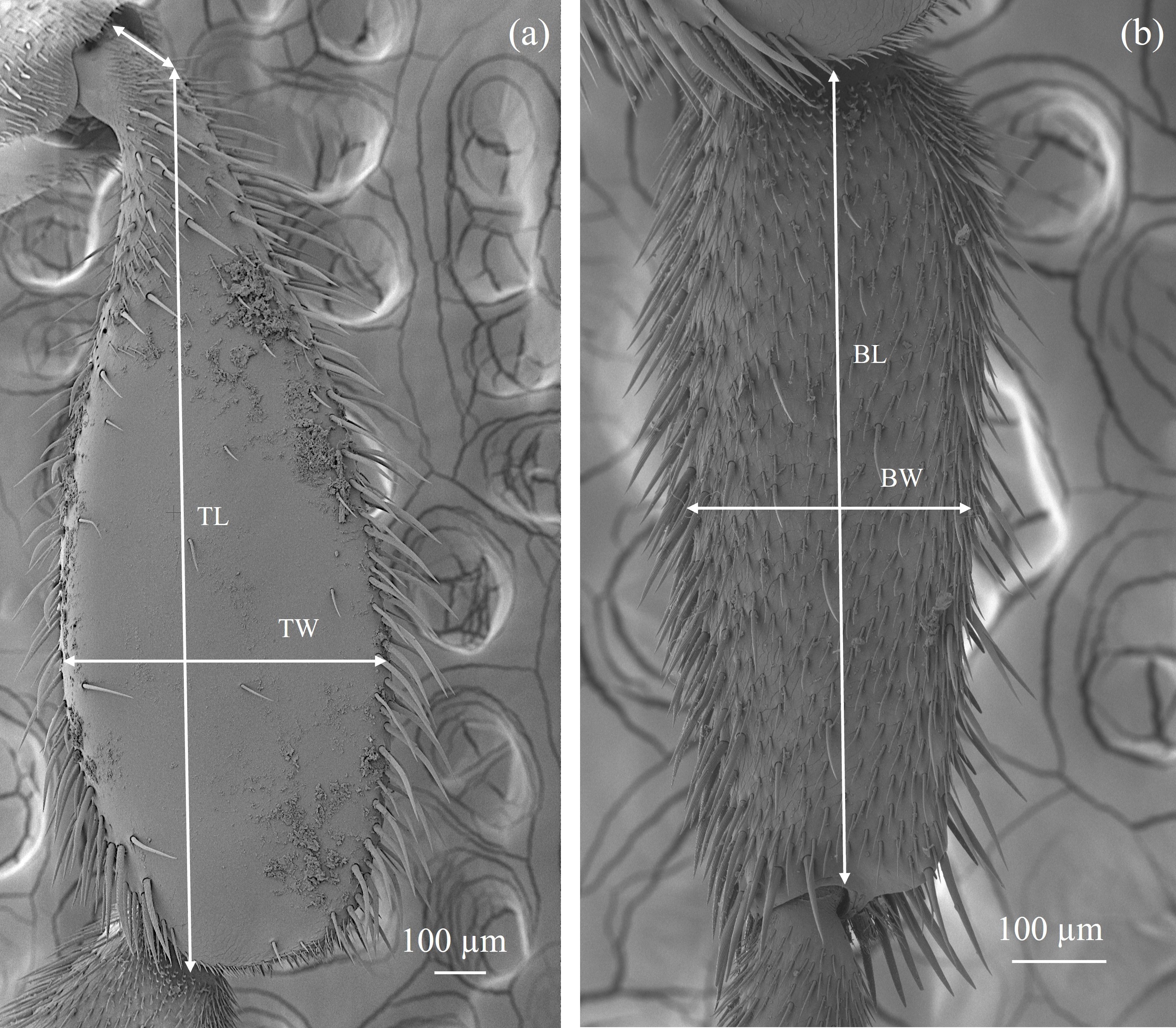

Supplement: Supplemental Information 6 — (a) TL: Tibia length and TB: Tibia breadth. (b) BL: Basitarsus length and BW: Basitarsus breadth. Tibia and basitarsus are of Lestrimelitta sp. [file peerj-13-19749-s006.jpg]

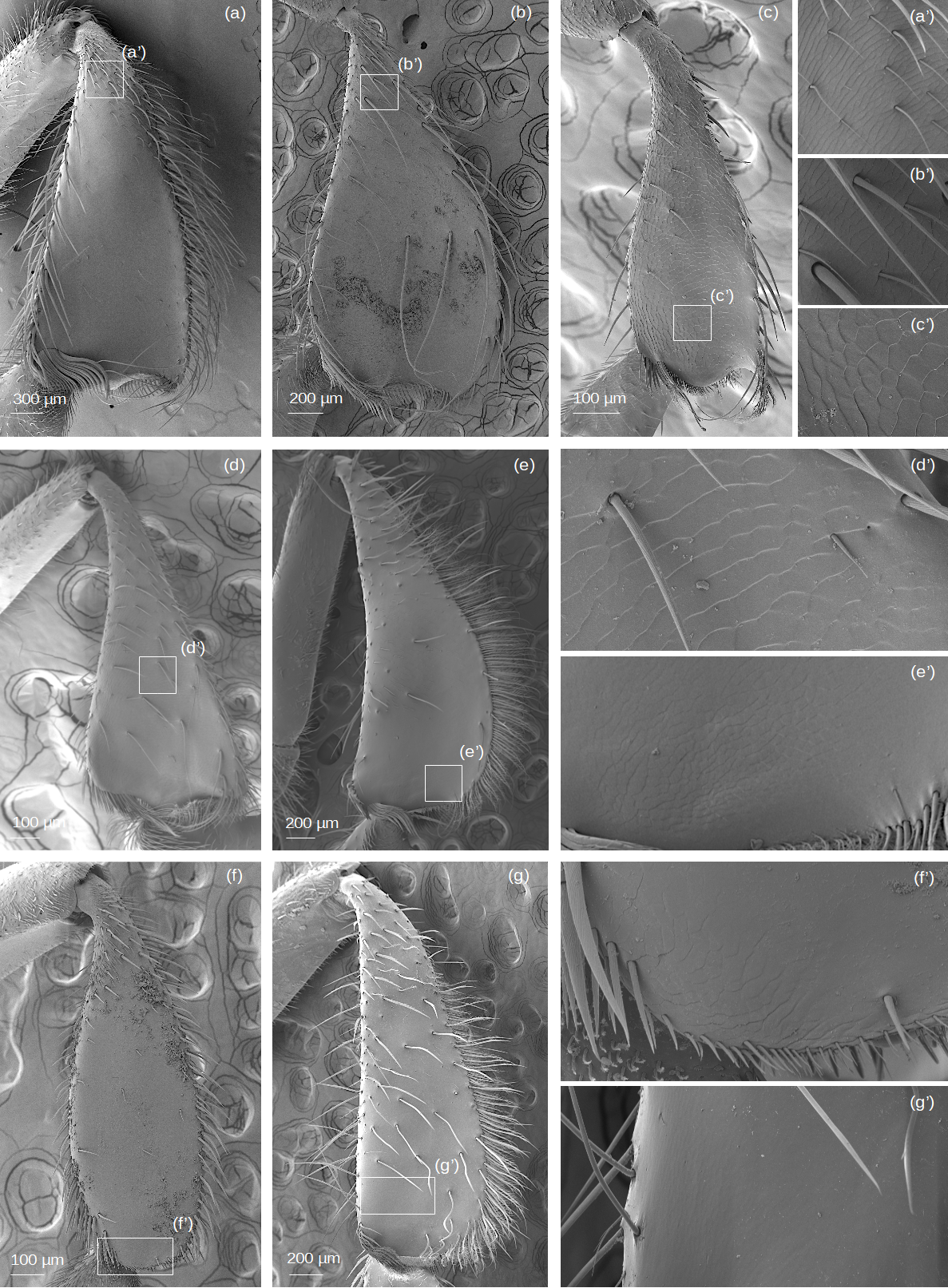

Supplement: Supplemental Information 7 — (A) M. cf. eburnea. (B) P. testacea. (c) T. cf. atomaria. (d) S. cf. latitarsis. (e) T. dallatorreana. (f) Lestrimelitta sp. and (g) T. cf. hypogea. (a’)(b’)(c’)(d’)(e’)(f’)(g’) Details of cuticular sculptures. [file peerj-13-19749-s007.png]

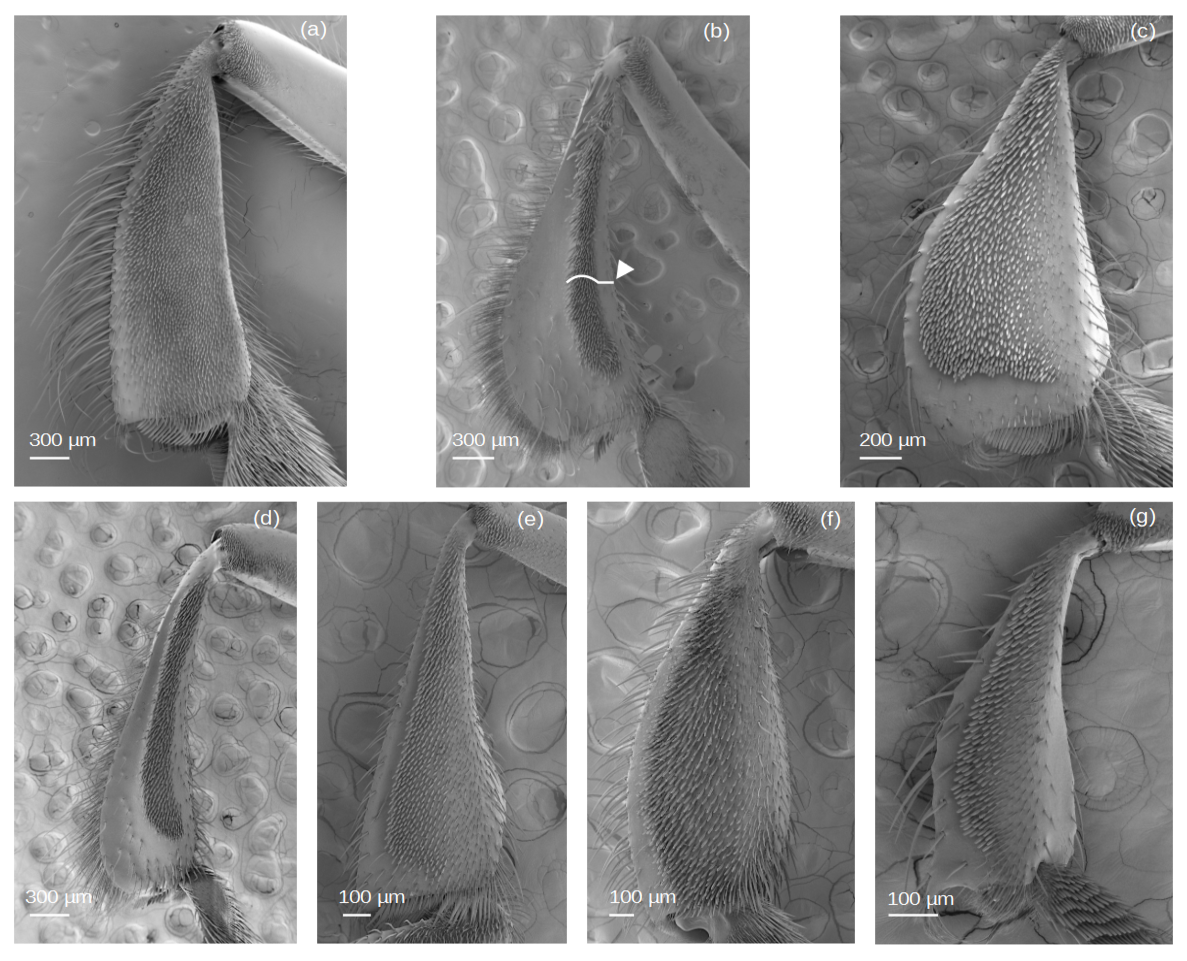

Supplement: Supplemental Information 8 — (A) M. cf. eburnea. (B) T. dallatorreana. The line in white indicated by the arrow shows the elevated area (clivulus) covered by keirotrichia. (c) P. testacea. (d) T. cf. hypogea. (e) S. cf. latitarsis. (f) Lestrimelitta sp..(g) T. cf. atomaria. [file peerj-13-19749-s008.png]

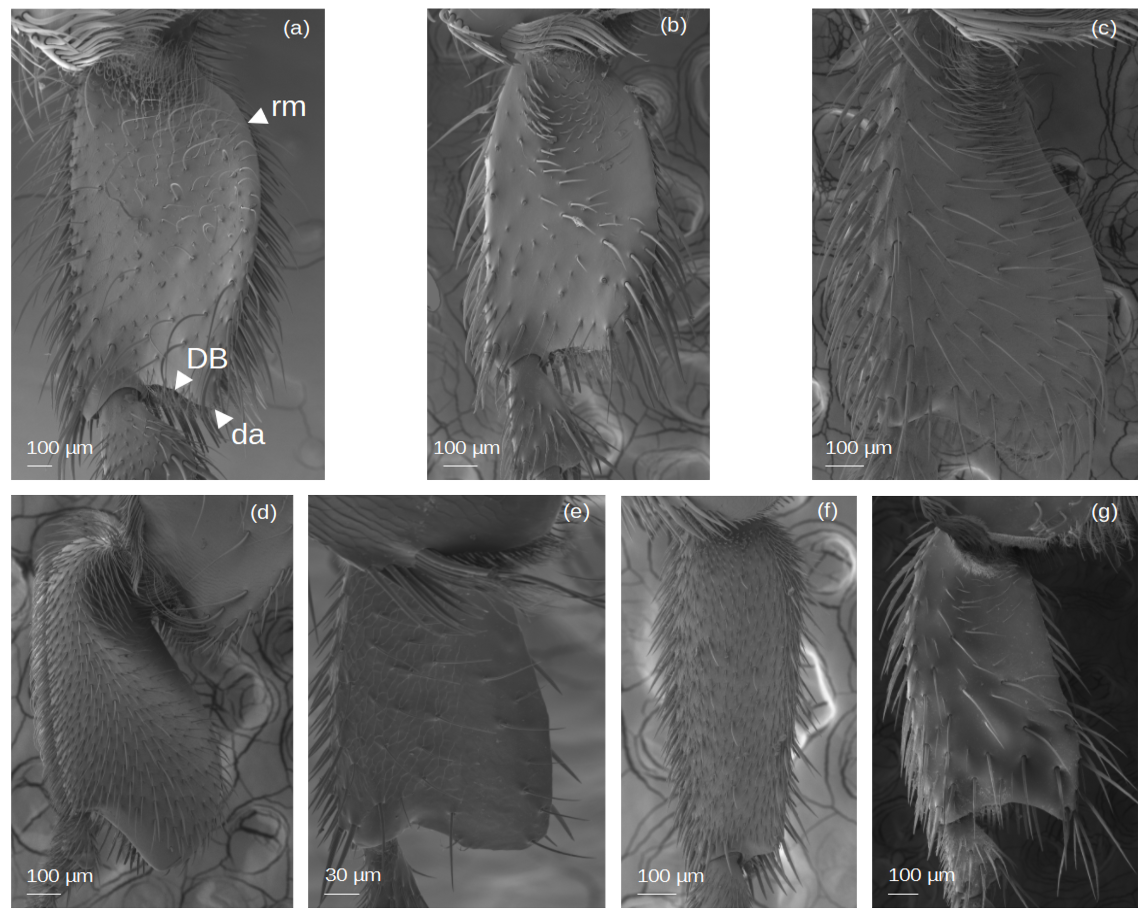

Supplement: Supplemental Information 9 — (A) M. cf. eburnea. (B) T. dallatorreana. (c) P. testacea. (d) S. cf. latitarsis. (e) T. cf. atomaria. (f) Lestrimelitta sp..(g) T. cf. hypogea. da: distal angle. DB: distal margin; rm: retrodorsal margin. [file peerj-13-19749-s009.png]

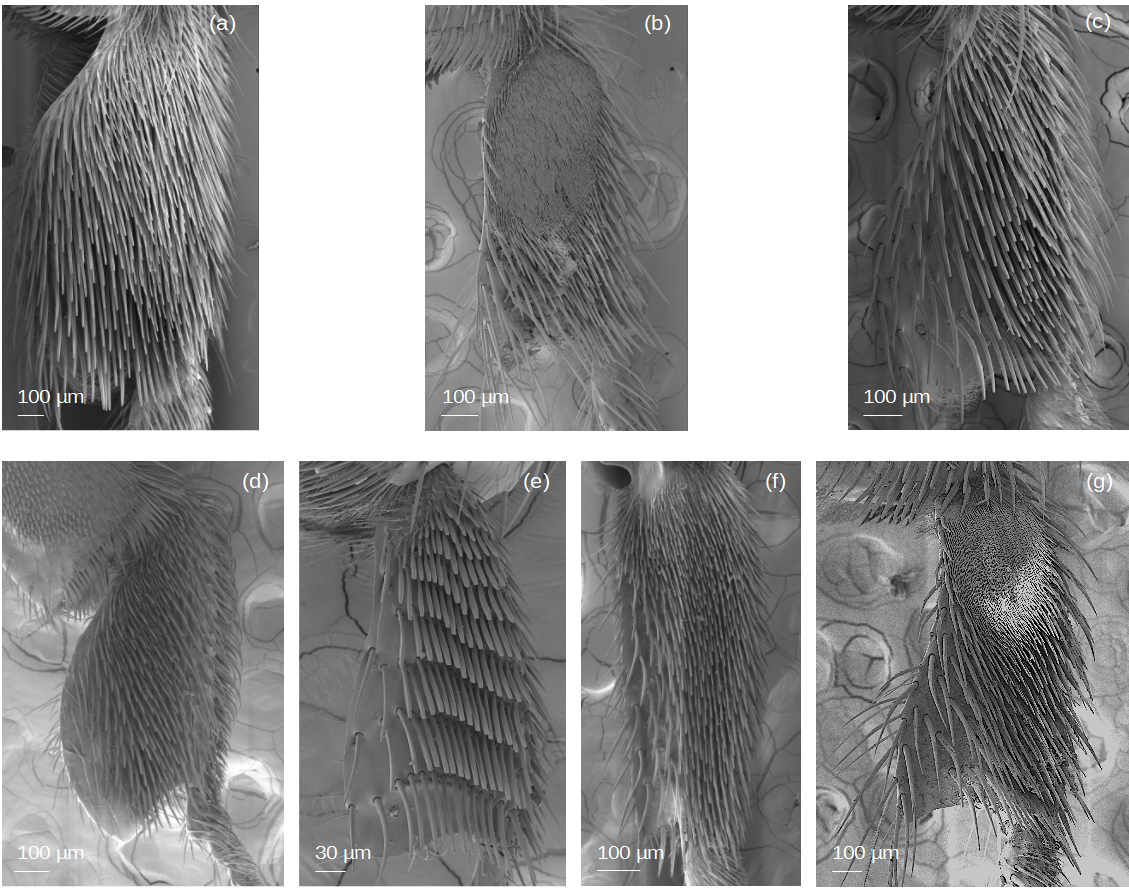

Supplement: Supplemental Information 10 — (A) M. cf. eburnea. (B) T. dallatorreana. (c) P. testacea. (d) S. cf. latitarsis. (e) T. cf. atomaria. (f) Lestrimelitta sp.. (g) T. cf. hypogea [file peerj-13-19749-s010.png]
